# Dihydrocapsaicin Attenuates Plaque Formation through a PPARγ/LXRα Pathway in apoE^−/−^ Mice Fed a High-Fat/High-Cholesterol Diet

**DOI:** 10.1371/journal.pone.0066876

**Published:** 2013-06-26

**Authors:** Yan-Wei Hu, Xin Ma, Jin-Lan Huang, Xin-Ru Mao, Jun-Yao Yang, Jia-Yi Zhao, Shu-Fen Li, Yu-Rong Qiu, Jia Yang, Lei Zheng, Qian Wang

**Affiliations:** 1 Laboratory Medicine Center, Nanfang Hospital, Southern Medical University, Guangzhou, Guangdong, China; 2 Department of Anesthesiology, Nanfang Hospital, Southern Medical University, Guangzhou, Guangdong, China; Max Delbrueck Center for Molecular Medicine, Germany

## Abstract

**Aims:**

Atherosclerosis is a chronic inflammatory disease and represents the major cause of cardiovascular morbidity and mortality. There is evidence that dihydrocapsaicin (DHC) can exert multiple pharmacological and physiological effects. Here, we explored the effect of DHC in atherosclerotic plaque progression in apoE^−/−^ mice fed a high-fat/high-cholesterol diet.

**Methods and Results:**

apoE^−/−^ mice were randomly divided into two groups and fed a high-fat/high-cholesterol diet with or without DHC for 12 weeks. We demonstrated that cellular cholesterol content was significantly decreased while apoA1-mediated cholesterol efflux was significantly increased following treatment with DHC in THP-1 macrophage-derived foam cells. We also observed that plasma levels of TG, LDL-C, VLDL-C, IL-1β, IL-6, TNF-α and CRP were markedly decreased while plasma levels of apoA1 and HDL-C were significantly increased, and consistent with this, atherosclerotic lesion development was significantly inhibited by DHC treatment of apoE^−/−^ mice fed a high-fat/high-cholesterol diet. Moreover, treatment with both LXRα siRNA and PPARγ siRNA made the up-regulation of DHC on ABCA1, ABCG1, ABCG5, SR-B1, NPC1, CD36, LDLR, HMGCR, apoA1 and apoE expression notably abolished while made the down-regulation of DHC on SRA1 expression markedly compensated. And treatment with PPARγ siRNA made the DHC-induced up-regulation of LXRα expression notably abolished while treatment with LXRα siRNA had no effect on DHC-induced PPARγ expression.

**Conclusion:**

These observations provide direct evidence that DHC can significantly decrease atherosclerotic plaque formation involving in a PPARγ/LXRα pathway and thus DHC may represent a promising candidate for a therapeutic agent for the treatment or prevention of atherosclerosis.

## Introduction

Coronary atherosclerosis represents the leading cause of morbidity and mortality in men and women throughout industrialized societies. Hypercholesterolemia, particularly of low-density lipoprotein (LDL) cholesterol and very low-density lipoprotein (VLDL) cholesterol, is a well-established risk factor for the development of atherosclerosis and its pathologic complications [1], [2]. It is now well accepted that atherosclerosis is not only a lipid disorder, but also a chronic inflammatory disease. Inflammatory processes are involved at all stages of the atherosclerotic development, from lesion initiation to plaque rupture [3], [4]. Therefore, factors that act to lower levels of cholesterol and to limit inflammation in this setting may prove to be beneficial in reducing disease progression.

Capsaicin, a pungent complex of related components known as capsaicinoids, is found in red peppers of the *Capsicum* genus and has been used as a spice, a food additive, and a drug [5]. The major components of capsaicinoids are capsaicin and dihydrocapsaicin (DHC), which together typically represent 85–90%of the total capsaicinoid content in pepper extract, and its minor components include nordihydrocapsaicin, homocapsaicin and homodihydrocapsaicin [6]. It has been shown that capsaicin is able to stimulate the release of calcitonin gene-related peptide (CGRP) by activating transient receptor potential channel vanilloid type 1 (TRPV1) and therefore it has potential benefits for cardiovascular function [7]. Adams *et al.* have demonstrated that capsaicin and DHC could inhibit platelet aggregation and the activity of clotting factors VIII and IX, a property which may contribute to the prevention of the onset and/or reduction of the incidence of cardiovascular diseases [8]. In addition, capsaicinoids can also contribute to their beneficial effects on the cardiovascular system through their antioxidant properties [9], [10]. These reports support the notion that capsaicinoids have potential beneficial effects on the prevention of cardiovascular diseases, such as atherosclerosis and coronary heart disease in particular.

In the present study, we aim to explore the impact of DHC on cholesterol metabolism and inflammatory gene expression, and to study the effect of DHC on plasma lipoprotein profiles, circulating cytokine levels, hepatic lipid deposition and the progression and regression of atherosclerosis in the apoE^−/−^ mice. Our findings demonstrate DHC administration could inhibit atherosclerotic lesion development in apoE^−/−^ mice fed a high-fat/high- cholesterol diet. A series of protein involved in cholesterol metabolism and inflammatory processes could be regulated by DHC through a PPARγ/LXRα pathway *in vivo* and *in vitro.*

## Materials and Methods

### Materials

Dihydrocapsaicin (N-[(4-hydroxy-3-methoxyphenyl)methyl]-8-methyl-6-nonanamide) was purchased from Sigma Chemical Company (St. Louis, MO, USA). The PrimeScript RT Reagent Kit (Perfect Real Time) (DRR037A) (TaKaRa, Japan), SYBR® Premix Ex TaqTM II (Tli RNaseH Plus) (DRR820A) (TaKaRa, Japan) were obtained as indicated. All other chemicals were of the best grade available from commercial sources.

### Animals and Diets

The investigation conforms with the Guide for the Care and Use of Laboratory Animals published by the US National Institutes of Health (NIH Publication No. 85-23, revised 1996) and was approved by the Animal Experimental Committee at Nanfang Hospital. Male 8-week-old apoE−/− mice in a C57BL/6 background (purchased from the Laboratory Animal Center of Peking University, China) were randomized into eight groups (control group (n = 20), DHC group (n = 20), si-Mock group (n = 20), si-Mock+DHC group (n = 20), si-LXRα group (n = 20), si-LXRα+DHC group (n = 20), si-PPARγ group (n = 20) and si-PPARγ+DHC group (n = 20)) and housed five per cage at 25°C on a 12-h light/dark cycle. All the mice were fed a high-fat/high-cholesterol diet containing 15% fat and 0.25% cholesterol (obtained from the Laboratory Animal Center of Peking University, China). The control and DHC groups were treated with either vehicle (cholesterol-free vegetable oil) or DHC (3.0 mg/kg body weight) daily by oral gavage (0.2 mL per mouse) for 12 weeks. The si-Mock group, si-PPARγ group and si-LXRα group were injected via the tail vein with control lentivirus (si-Mock), with lentivirus encoding mouse PPARγ (si-PPARγ) or with lentivirus encoding mouse LXRα (si-LXRα) respectively and then treated with cholesterol-free vegetable oil daily by oral gavage (0.2 mL per mouse) for 12 weeks. The si-Mock+DHC group, si-LXRα+DHC group and si-PPARγ+DHC group were injected via the tail vein with control lentivirus (si-Mock+DHC), with lentivirus encoding mouse LXRα (si-LXRα+DHC) or with lentivirus encoding mouse PPARγ (si-PPARγ+DHC) respectively and then treated with DHC (3.0 mg/kg body weight) daily by oral gavage (0.2 mL per mouse) for 12 weeks. Mice were administered by gavage under light ether anaesthesia each day and body weight was monitored at regular intervals. At week 12, mice were anaesthetized with inhaled methoxyflurane, 1 mL of blood was collected by cardiac puncture before mice were sacrificed by cervical dislocation and tissues were collected for further analysis. The adequacy of anaesthesia was monitored by testing tactile stimulus response and forelimb or hindlimb pedal withdrawal reflex, and continual observation of respiratory pattern, mucous membrane color, and responsiveness to manipulations throughout the procedure.

### Preparation of Ox-LDL

Native LDL was purchased from Sigma. Native LDL (200 *µ*g protein/ml) was oxidized by exposure to CuSO4 (5 µmol/l free Cu^2+^) in phosphate-buffered saline (PBS) at 37°C for 24 hours. Control incubations were treated with 200 µmol/l EDTA without CuSO4. The freshly prepared ox-LDL was dialyzed at 4°C for 48 h against 500 volumes of PBS to remove Cu^2+^ and was sterilized by passage through a 0.45 µm filter. Oxidation of LDL was confirmed by the measurement of thiobarbituric acid-reactive substances (TBARS) with malonaldehyde bis (dimethyl acetal) (MDA) as the standard. The TBARS content of ox-LDL was 6.05±0.16 versus 0.32±0.15 nmol/100 µg protein in the native LDL preparation (p<0.01). Protein content was determined by bicinchoninic acid (BCA) protein assay kit (Pierce, Rockford, USA) with the use of bovine serum albumin (BSA) as the standard. The ox-LDL was kept in 50 mmol/L Tris-HCl, 0.15 mol/L NaCl and 2 mmol/L EDTA at pH 7.4 and was used within ten days of preparation.

### Cell Culture

Human monocytic THP-1 cells, HepG2 cells and Caco-2 cells were obtained from American Type Culture Collection (ATCC, Manassas, VA, USA). THP-1 cells were maintained in RPMI 1640 medium containing 10% fetal calf serum (FCS) in the presence of streptomycin (100 µg/mL), penicillin (100 U/mL) and differentiated for 72 h with 100 nM phorbol 12-myristate 13-acetate (PMA). Macrophages were transformed into foam cells by incubation in the presence or absence of 50 *µ*g/mL ox-LDL in serum-free RPMI1640 medium containing 0.3% BSA for 48 h. HepG2 cells and Caco-2 cells were grown in Dulbecco’s modified Eagle’s medium (DMEM) containing 10% FCS with streptomycin (100 µg/mL) and penicillin (100 U/mL). All cells were incubated at 37°C, 5% CO_2_. Cells were seeded in 6- or 12-well plates or 60-mm dishes and grown to 80–90% confluence before use.

### Cytokine Assays and Measurement of Serum Biochemical Parameters

The levels of human TNF-α, IL-1β, TGF-β and IL-6 present in the culture media (R&D Systems, Minneapolis, MN, USA), the serum concentrations of IL-1β, IL-6 and TNF-α (R&D Systems, Minneapolis, MN, USA), the serum CRP amount (Diagnostic System Laboratories, Webster, TX, USA) and the levels of serum apolipoprotein A1 and apolipoprotein B100 (Cusabio Biotech Co., Ltd., China) were measured by ELISA according to the manufacturer’s instructions. The T-Cho, TG, LDL-C, HDL-C and VLDL-C concentrations were determined enzymatically using an automated analyzer.

### RNA Isolation and Real-time Quantitative PCR Analysis

Total RNA from mouse tissues or cultured cells was extracted using TRIzol reagent (Invitrogen) in accordance with the manufacturer’s instructions. Real-time quantitative PCR, using SYBR Green detection chemistry, was performed on an ABI 7500 Fast Real Time PCR system (Applied Biosystems, Foster City, CA, USA). Melt curve analyses of all real-time PCR products were performed and shown to produce a single DNA duplex. All samples were measured in triplicate and the mean value was considered for comparative analysis. Quantitative measurements were determined using the ΔΔCt method and GAPDH expression was used as the internal control. Primer sequences are given in Table 1 and Table 2.

**Table 1:** Primer sequences for human mRNAs measured by real-time PCR (The primer sequences are listed from 5′ to 3′).

mRNA	Forward Primer	Reverse Primer
ABCA1	GTCCTCTTTCCCGCATTATCTGG	AGTTCCTGGAAGGTCTTGTTCAC
ABCG1	TCTTCGTCAGCTTCGACACCA	TCTCGTCGATGTCACAGTGCAG
SR-B1	ATGAAATCTGTCGCAGGCATTG	TGCATCACCTTGGGCATCA
NPC1	AGCCACATAACCAGAGCGTTCAC	CCATGGCCAAATACATCCTGAAG
CAV-1	CCTCAACGATGACGTGGTCAA	TCGTCACAGTGAAGGTGGTGAAG
SRA1	TTTGGAACAGGCATTGGAAG	GCGGTGGATGTCATCTGCT
CD36	GAGAACTGTTATGGGGCTAT	TTCAACTGGAGAGGCAAAGG
LDLR	GGCAGTGTGACCGGGAATATG	TTCGCCGCTGTGACACTTG
ABCG5	CCTTGACAGGCACTCAAATG	TTTCTCAATGAATTGAATTCCTT
NPC1L1	GGGTGGATGACTTCATTGACTGG	CATCGTGATGCTCATGCAGTTC
MTP	GCAGATGGACAAGGATGAAGCTC	GCGGGAATTCACATCCTGCTA
apoA1	ACTGTGTACGTGGATGTGCTCAAAG	CACGCTGTCCCAGTTGTCAAG
CAV-1	CCTCAACGATGACGTGGTCAA	TCGTCACAGTGAAGGTGGTGAAG
apoE	TCTGAGCAGGTGCAGGAGGA	GTTGTTCCTCCAGTTCCGATTTGTA
HMGCR	GCCTGGCTCGAAACATCTGAA	CTGACCTGGACTGGAAACGGATA
HMGS	CAGCTGCTGTCTTCAATGCTGTTA	AGCTACTGCTCCAACTCCACCTG
LXRα	TCTGGAGACATCTCGGAGGTACAAC	AGCAAGGCAAACTCGGCATC
PPARγ	TGGAATTAGATGACAGCGACTTGG	CTGGAGCAGCTTGGCAAACA
SREBP2	CAAGGCCCTGGAAGTGACA	AGGAACTCTGCTGCCCATCTG
SREBP-1c	CTCCGGCCACAAGGTACACA	GAGGCCCTAAGGGTTGACACAG
CRP	AATGTGAACATGTGGGACTTTGTG	CGCCAGTTCAGGACATTAGGAC
TNF-α	CACTCCAGCAGCTCAAGCAGA	GTGCACCAGCTCAATGGTTTC
TGF-β1	GCGACTCGCCAGAGTGGTTA	GTTGATGTCCACTTGCAGTGTGTTA
IL-1β	CCAGGGACAGGATATGGAGCA	TTCAACACGCAGGACAGGTACAG
IL-6	AAGCCAGAGCTGTGCAGATGAGTA	TGTCCTGCAGCCACTGGTTC
NF-κ B	GCCTCCACAAGGCAGCAAATA	CACCACTGGTCAGAGACTCGGTAA
PPARα	ACTTATCCTGTGGTCCCCGG	CCGACAGAAAGGCACTTGTGA
PPARδ	TCATTGCGGCCATCATTCTGTGTG	TTCGGTCTTCTTGATCCGCTGCAT

**Table 2:** Primer sequences for mouse mRNAs measured by real-time PCR (The primer sequences are listed from 5′ to 3′).

mRNA	Forward Primer	Reverse Primer
ABCA1	TGAAGCCTGTCCAGGAGTTC	ATGACAAGGAGGATGGAAGC
ABCG1	CAAGACCCTTTTTGAAAGGGATCTC	GCCAGAATATTCATGAGTGTGGAC
LXRα	TGACTTTGCCAAACAGCTC	AGCATGACTCGATTGCAGAG
PPARγ	AGGACATCCAAGACAACCTGC	TCTGCCTGAGGTCTGTCATC
TNF-α	TCTTCTGTCTACTGAACTTCG	GAAGATGATCTGAGTGTGAGG
IL-1β	CAACCAACAAGTGATATTCTCCATG	GATCCACACTCTCCAGCTGCA
IL-6	CTGCAAGAGACTTCCATCCAGTT	GAAGTAGGGAAGGCCGTGG
NF-κ B	ACCACTGCTCAGGTCCACTGTC	GCTGTCACTATCCCGGAGTTCA
MCP-1	CAGCCAGATGCAGTTAACG	TCTCTCTTGAGCTTGGTGAC
MIP-1α	ACCTGGAACTGAATGCCTGAGA	GCTTATAGGAGATGGAGCTATGCA
ICAM-1	AACTGTGGCACCGTGCAGTC	AGGGTGAGGTCCTTGCCTACTTG
VCAM-1	GCCACCCTCACCTTAATTGCTATG	TGTGCAGCCACCTGAGATCC

### Western Blot Analyses

Cells were harvested and protein extracts prepared according to Instruction Manual. Extracts were then subjected to Western blot analyses [10% SDS-polyacrylamide (SDS-PAGE); 30 *µ*g protein per lane] using rabbit polyclonal anti-LXRα, -SRA1, -CD36, -GRα, -SREBP1c, -SREBP2, -PPARα, -PPARδ antibodies (Proteintech group, Inc., Chicago, IL, USA), rabbit polyclonal anti-PPARγ, -ABCA1 antibodies (Abcam Inc., Cambridge, MA), rabbit polyclonal anti-TRα, -HNF-4α antibodies (Bioworld Technology., Minneapolis, USA), rabbit polyclonal anti-ABCG1, -SR-B1, -NPC1, -LDLR, -CAV-1, -HNF-1α, -LRH1, -Foxa2, -apoA1, -HMGCR, -HMGS, -apoE, -NPC1L1, -ABCG5 antibodies (Epitomics., CA, USA) and rabbit polyclonal anti -apoA1, -HMGS, -ABCG5, -MTP, -β-actin antibody (Santa, Cruz, CA, USA). The proteins were visualized using a chemiluminescence method (ECL Plus Western Blot Detection System; Amersham Biosciences, Foster City, CA, USA).

### Transfection with siRNA

The siRNAs against LXRα (LXRα-siRNA, anti-sense strand, 5′-CUGCCCAGCAACAGUGUAA dTdT-3′, sense strand, 3′ dTdT GACGGGUCGUUGUCACAUU-5′) and PPARγ (PPARγ-siRNA, anti-sense strand, 5′- GUACCAAAGUGCAAUCAAA dTdT-3′, sense strand, 3′- dTdT CAUGGUUUCACGUUAGUUU-5′) and an irrelevant 21-nucleotide control siRNA (Negative Control) were purchased from Ribo Biotechnology. Cells (2×10^6^ cells/well) were transfected using Lipofectamine 2000 (Invitrogen). 48 h after transfection, real-time RT-PCR and western blots were performed.

### Lentivirus Production and Tail Vein Injection

Three pairs of double-strand DNA targeting mouse PPARγ or LXRα mRNA and one pair of negative control double-strand DNA were designed, synthesized and cloned into lentiviral vector pGCSIL-GFP to generate pGCSIL-GFP-shPPARγ lentvirus (si-PPARγ), pGCSIL-GFP-shLXRα lentvirus (si-LXRα) and the control lentvirus named pGCSIL-GFP-NC (si-Mock) respectively. Viral multiplicity of infection for liver infection was estimated based on in vitro primary hepatocyte transduction efficiency: 0.5 mL of undiluted viral stocks supplemented with Polybrene (7.5 µg/mL) was added to 10^5^ primary hepatocytes cultured in 12-well plates, and green fluorescent protein (GFP)-positive cells were counted 96 hours after transduction. Anesthetized C57BL/6 mice and apoE^−/−^ mice were injected with 150 µL of undiluted viral stocks supplemented with Polybrene (5 µg/mL) into the tail vein. C57BL/6 mice and apoE^−/−^ mice were respectively anaesthetized and sacrificed at 7 days and 12 weeks by cervical dislocation and tissues were collected for further analysis.

### Transcriptional Activity Assay

Transcription activity of PPARα, PPARγ and LXRα was assayed using an enzyme-linked immunosorbent assay-based PPARα, PPARγ or LXRα Transcription Factor Assay Kit (Cayman Chemical, Cayman Chemical and Active Motif Inc, respectively). Sample proteins were extracted from cells according to the manufacturer's instructions, and were added to a 96-well plate that had been immunobilized by an oligonucleotide containing PPARα response element, PPARγ response element or LXRα response element. After 1 h, the wells were incubated with diluted primary PPARα antibody, primary PPARγ antibody or primary LXRα antibody. The horseradish eroxidase-conjugated secondary antibody was added and incubation conducted for 1 h. At the end, the reaction was stopped, and absorbance was read at 450 nm on a spectrophotometer.

### DiI-labeled ox-LDL Uptake Assays

PMA-differentiated THP-1 cells were treated with DHC (100 µM) for 24 h, after which fluorescent-tagged Dil-oxLDL was added, and the cells were incubated for 24 h. Adherent cells were harvested and washed three times with phosphate buffer saline (PBS). Analysis was performed on a fluorescent activated cell sorting (FACS) calibur flow cytometer (Becton Dickinson, Franklin Lakes, NJ, USA) with Cell Quest Pro software (BD Biosciences, San Jose, CA, USA).

### Cellular Cholesterol Assays

High performance liquid chromatography (HPLC) analysis was conducted as previously described [11]. The sterol analyses were performed using a HPLC system (model 2790, controlled with Empower Pro software; Waters Corp., Milford, MA, USA). Absorbance at 216 nm was monitored. Data were analyzed with TotalChrom software from PerkinElmer (Waltham, MA, USA).

### Cellular Cholesterol Efflux Experiments

Cells were cultured and treated with DHC (100 µM) for 24 h before being labeled with 0.2 µCi/mL [^3^H]cholesterol. After 72 h, cells were washed with PBS and incubated overnight in RPMI 1640 medium containing 0.1% (w/v) BSA to allow equilibration of [^3^H]cholesterol in all cellular pools. Equilibrated [^3^H]cholesterol-labeled cells were washed with PBS and incubated in 2 mL of efflux medium containing RPMI 1640 medium and 0.1% BSA with 25 µg/mL human plasma apoA1. A 150 µL sample of efflux medium was obtained at the times designated and passed through a 0.45-µm filter to remove any floating cells. Monolayers were washed twice with PBS and cellular lipids were extracted with isopropanol. Medium and cell-associated [^3^H]cholesterol was then measured by liquid scintillation counting. Percent efflux was calculated by the following equation: [total media counts/(total cellular counts+total media counts)]×100%.

### En Face Plaque Area

Immediately after mice were killed, the aorta was excised and fixed in 10% buffered formalin for quantification of en face plaque area. Briefly, after the adventitial tissue was carefully removed, the aorta was opened longitudinally, stained with Oil Red O (Sigma), and pinned on a blue wax surface. En face images were obtained by a stereomicroscope (SZX12, Olympus, Tokyo) equipped with a digital camera (Dxm1200, Nicon, Tokyo) and analyzed using Adobe Photoshop version 7.0 and Scion Image software. The percentage of the luminal surface area stained by Oil Red O was determined [12].

### Quantification of Atherosclerosis in the Aortic Sinus

The upper portion of the heart and proximal aorta were obtained, embedded in Optimal Cutting Temperature (OCT) compound (Fisher, Tustin, CA), and stored at –70°C. Serial 10-µm thick cryosections of aorta, beginning at the aortic root, were collected for a distance of 400 µm. Sections were stained with Oil Red O. The Oil Red O -positive areas in digitized color images of stained aortic root sections were quantified using Image-Pro Plus image analysis software (Media Cybernetics), and the data are expressed as percent of total section area.

### Liver Histology and Oil Red O Staining

To examine liver morphology, formalin-fixed paraffin embedded sections of liver were stained with hematoxylin and eosin (H&E). Hepatic lipid deposition was assessed in samples collected in optimal cutting temperature compound (OCT) by Oil Red O staining. Briefly, liver cryosections were fixed for 10 min in 60% isopropanol and stained with 0.3% Oil Red O in 60% isopropanol for 30 min and subsequently washed with 60% isopropanol. Sections were counterstained with Gill’s hematoxylin, washed with acetic acid solution (4%), and mounted with aqueous solution. The area of positive staining for Oil Red O was calculated as a percentage of total section area, and an average lipid droplet size was calculated by utilizing ImagePro Plus (Media Cybernetics) from five views per animal.

### Statistical Analyses

Data are expressed as means ± S.D. The data were compared by one way ANOVA followed by Student- Newman- Keuls (SNK) test or unpaired Student's *t* test, using Graph-Pad Prism (GraphPad Software, Inc.). Statistical significance was obtained when *p* values were less than 0.05.

## Results

### Effect of DHC on Lipid Loading, Lipid Content and Cholesterol Efflux

**Figure 1:**
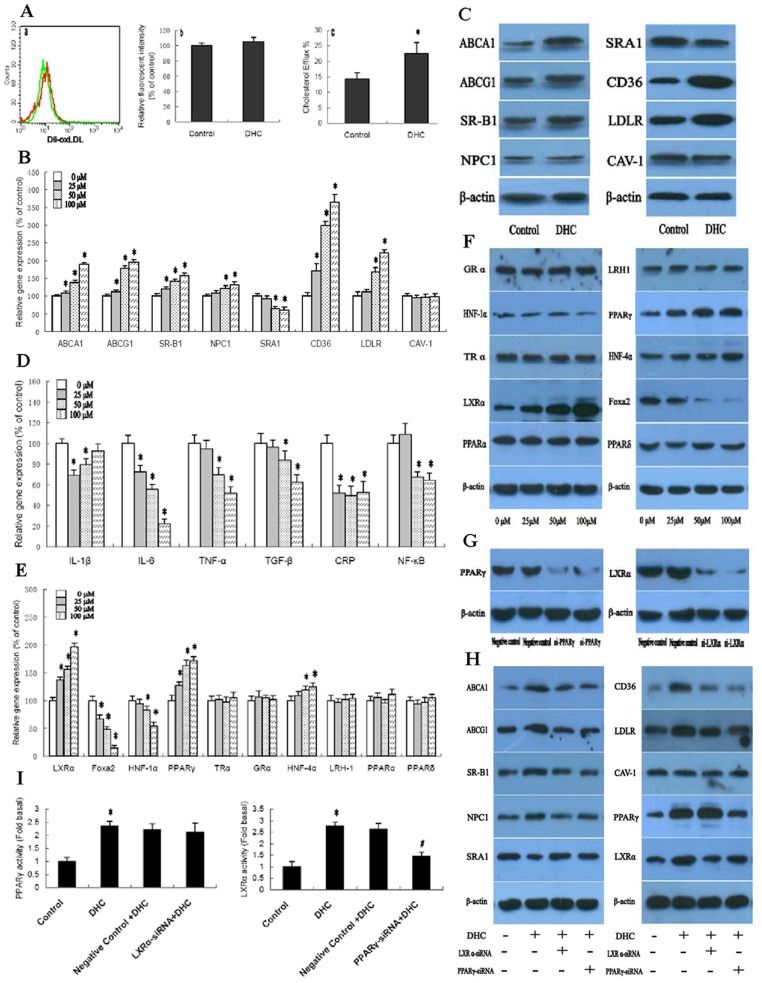
Effect of DHC on lipid loading, lipid content and cholesterol efflux. (A) a and b, THP-1 macrophages were treated with 0 µM DHC (control) or 100 µM DHC (DHC) for 24 h, and then incubated with 5 µg/mL DiI-labeled ox-LDL for 24 h. Uptake of DiI-labeled ox-LDL was analyzed by flow cytometry. a, Representative histogram of DiI-ox-LDL uptake in the presence of 100 µM DHC (green peak) or 0 µM DHC (red peak). b, DiI-ox-LDL uptake did not change in THP-1 macrophages treated with DHC compared to controls (p>0.05). c, THP-1 macrophage-derived foam cells were treated with 0 µM DHC (control) or 100 µM DHC (DHC) for 24 h, and then cellular cholesterol efflux was analyzed by liquid scintillation counting assays as shown above. (B, D, E and F) THP-1 macrophage-derived foam cells were treated with DHC as indicated for 24 h. B, D and E, Gene expression was measured by real-time quantitative PCR. F, protein expression was measured by western blot. (C) THP-1 macrophage-derived foam cells were treated with 0 µM DHC (control) or 100 µM DHC (DHC) for 24 h, and then protein expression was measured by western blot. (G) THP-1 macrophages were transfected with control or LXRα siRNA or PPARγ siRNA for 48 h. The protein expressions were measured by western blot. (H and I) The cells were transfected with control or LXRα siRNA or PPARγ siRNA, and then incubated with 100 µM DHC for 24 h. The protein expression and transcriptional activity were measured by western blot and Transcription Factor Assay Kit, respectively. All results are expressed as the mean ± S.D. of three independent experiments, each performed in triplicate. A, C and G, the data were compared by unpaired Student's t test. B, D, E, F, H and I, the data were compared by one way ANOVA followed by SNK test. *p<0.05 vs. control group.

We first investigated the role of DHC on lipid loading in THP-1 macrophages by flow cytometry. As shown (Fig. 1A), DiI-labeled ox-LDL uptake did not change following treatment with DHC. Next, we examined the effect of DHC on cholesterol content and cholesterol efflux in THP-1 macrophage-derived foam cells by high performance liquid chromatography and liquid scintillation counting assays. As shown, cellular cholesterol content (Table 3) was decreased while cholesterol efflux (Fig. 1A) was increased when cells were treated with DHC.

**Table 3:** Effect of DHC on cholesterol content in THP-1 macrophage-derived foam cells.

	Control	25 µM	50 µM	100 µM
TC (mg/dL)	469±35	441±33	316±30*	255±29*
FC (mg/dL)	181±21	171±17	129±19*	105±21*
CE (mg/dL)	286±27	270±25	187±23*	150±21*
CE/TC (%)	61.3	61.2	59.1	58.8

THP-1 macrophage-derived foam cells were divided into four groups and cultured in medium at 37°C containing 0 µM, 25 µM, 50 µM and 100 µM DHC for 24 h, respectively. Cellular cholesterol and cholesterol ester were extracted as described above. HPLC was performed to determine the cellular total cholesterol (TC), free cholesterol (FC) and cholesterol ester (CE). The results are expressed as the mean ± S.D. of six independent experiments, each performed in triplicate. The data were compared by one way ANOVA followed by SNK test.

* *p*<0.05 vs. control group.

Subsequently, we aimed to explore the mechanism underlying the altered cellular lipid profile in THP-1 macrophage-derived foam cells following treatment with DHC. For this purpose, we performed gene and protein expression of aBCA1, ABCG1, SR-B1, NPC1, CD36, LDLR, SRA1 and CAV-1 in THP-1 macrophage-derived foam cells. As shown (Figs. 1B and 1C), transcript levels and protein levels of ABCA1, ABCG1, SR-B1, NPC1, CD36 and LDLR were up-regulated by treatment with DHC. In contrast, expression of SRA1 was down-regulated by DHC treatment. However, DHC has no effect on CAV-1 expression in THP-1 macrophage-derived foam cells.

Atherosclerosis is indeed a complex inflammatory disease and the macrophage foam cell is the major cell type involved in this disease [13], [14]. Therefore, we next investigated the effect of DHC on inflammatory gene expression in THP-1 macrophage-derived foam cells. As shown in Fig. 1D, gene levels of IL-6, TNF-α, TGF-β, and NF-κB were down-regulated by treatment with DHC in a dose-dependent manner. Moreover, gene expression of CRP and IL-1β could also be down-regulated by treatment with DHC in a dose-independent manner. The concentrations of four inflammatory cytokines were monitored in THP-1 macrophage-derived foam cells after treatment with DHC for 48 h. There were marked decreases in IL-6 level during the observed time period. However, only modest differences were observed for IL-1β, TNF-α and TGF-β (Table 4).

**Table 4:** Effect of DHC on inflammatory cytokines in THP-1 macrophage-derived foam cells.

	IL-6 (ng/mL)	TNF-α(ng/mL)	TGF-β(ng/mL)	IL-1β(ng/mL)
Control	1.05±0.25	439±52	7.9±2.3	275±43
DHC	0.27±0.15*	312±39*	5.1±1.5*	249±32*

THP-1 macrophage-derived foam cells were divided into two groups and cultured in medium at 37°C containing 0 µM and 100 µM DHC for 24 h, respectively. The quantitation of secreted inflammatory cytokines was performed by ELISA. The results are expressed as the mean ± S.D. of six independent experiments, each performed in triplicate. The data were compared by unpaired Student's *t* test.

* *p*<0.05 vs. control group.

Nuclear receptors are a class of intracellular transcription factor activated by ligands. They play key roles in the inflammatory response and lipid metabolism via several mechanisms [15], [16]. Cellular and whole-body cholesterol homeostasis is maintained through a network of transcriptional programs [17], [18]. Thus, we explored the gene and protein expression of LXRα, Foxa2, HNF-1α, PPARγ, TRα, GRα, HNF-4α, LRH1, PPARα and PPARδ in THP-1 macrophage- derived foam cells. As shown in Figs. 1E and 1F, both transcript and protein levels of LXRα, PPARγ and HNF-4α could be significantly up-regulated by treatment with DHC in a dose-dependent manner. In the contrary, both transcript and protein levels of Foxa2 and HNF-1α could be significantly down-regulated by treatment with DHC in a dose-dependent manner. However, the gene and protein expression of TRα, GRα, LRH1, PPARα and PPARδ did not changed by treatment with DHC. We then examined the effect of LXRα siRNA and PPARγ siRNA on the regulation of ABCA1, ABCG1, SR-B1, NPC1, CD36, LDLR, SRA1 and CAV-1 which was induced by DHC (Fig. 1H). As shown (Fig. 1G), in comparison to the control siRNA, the siRNA of PPARγ suppressed the expression of PPARγ proteins by 87% and the siRNA for LXRα suppressed the expression of LXRα proteins by 85% in THP-1 macrophage-derived foam cells. Treatment with both LXRα siRNA and PPARγ siRNA made the up-regulation of DHC on ABCA1, ABCG1, SR-B1, NPC1, CD36 and LDLR expression notably abolished while made the down-regulation of DHC on SRA1 expression markedly compensated. Interestingly, treatment with PPARγ siRNA made the DHC-induced up-regulation of LXRα expression notably abolished while treatment with LXRα siRNA have no effect on DHC-induced PPARγ expression. Moreover, treatment with both LXRα siRNA and PPARγ siRNA had no effect on CAV-1 expression. In addition, we explored effect of DHC on transcriptional activity of PPARα, PPARγ and LXRα and examined its mechanism by using LXRα siRNA and PPARγ siRNA in THP-1 macrophage-derived foam cells. As shown (Fig. 1I), transcriptional activity of both PPARγ and LXRα were enhanced by treatment with DHC. However, transcriptional activity of PPARα could not be induced by treatment with DHC (data not shown). Compared with treatment with control siRNA, the DHC-induced increase of transcriptional activity of LXRα was restored by treatment with PPARγ siRNA. However, the DHC-induced increase of transcriptional activity of PPARγ could not be restored by treatment with LXRα siRNA when compared with control siRNA treatment. These results suggest that expressions of ABCA1, ABCG1, SR-B1, NPC1, CD36, LDLR and SRA1 could be regulated by DHC through a PPARγ/LXRα pathway.

### Effect of DHC on Plasma Lipid Parameters and Circulating Cytokine Levels

Because of the key role of DHC in cholesterol and lipid metabolism, we examined the terminal plasma lipid levels from experimental mice. As shown in Table 5, treatment of apoE^−/−^ mice fed a high-fat/high-cholesterol diet with DHC led to a moderate 15.2% decrease in plasma TG levels. Plasma HDL-C showed a moderate 42.3% increase in the DHC group compared to the control group. Concomitantly, plasma LDL-C and VLDL-C levels were reduced by 28.3% and 37.6% in the DHC group, respectively. In addition, treatment with DHC led to a 33.3% increase in plasma apoA1 compared to the control group. Also, treatment with DHC led to a 10.4% decrease in body weight compared to the control group. However, no significant alteration in apoB levels or total cholesterol occurred.

**Table 5:** Effect of DHC on Plasma Lipids and Lipoprotein Values in apoE^−/−^ Mice.

	Control (n = 10)	DHC (n = 10)
Body weight (g)	30.9±2.65	27.7±2.02*
TG (mmol/L)	1.27±0.32	1.07±0.25*
TC (mmol/L)	28.86±3.69	27.58±4.02
HDL-C (mmol/L)	9.86±1.11	14.03±2.01*
LDL-C (mmol/L)	18.12±2.57	13.00±2.29*
VLDL-C (mmol/L)	0.88±0.12	0.55±0.31*
ApoA1 (g/L)	0.06±0.01	0.08±0.01*
ApoB (g/L)	0.16±0.02	0.16±0.02

Data are expressed as mean±S.D. The data were compared by unpaired Student's *t* test.

* *p*<0.05 vs. control group.

To investigate whether DHC-mediated changes in cellular proinflammatory gene expression could result in the corresponding changes in plasma inflammatory cytokines, we conducted a series of ELISA tests (Table 6). Consistent with the inflammatory gene expression data in THP-1 macrophage-derived foam cells, treatment with DHC resulted in down-regulation of IL-1β, IL-6, TNF-α and CRP concentrations in plasma by 25.5%, 39.5%, 27.6 and 43.9%, respectively.

**Table 6:** Effect of DHC on Plasma Cytokine Levels in apoE^−/−^ Mice.

	Control (n = 10)	DHC (n = 10)
IL-1β (pg/mL)	12.6±3.27	9.39±2.17*
IL-6 (pg/mL)	65.56±5.46	39.66±4.73*
TNF-α (pg/mL)	11.43±3.29	8.28±2.34*
CRP (ng/mL)	45.62±5.66	25.59±4.63*

Data are expressed as mean±S.D. The data were compared by unpaired Student's *t* test.

* *p*<0.05 vs. control group.

**Figure 2:**
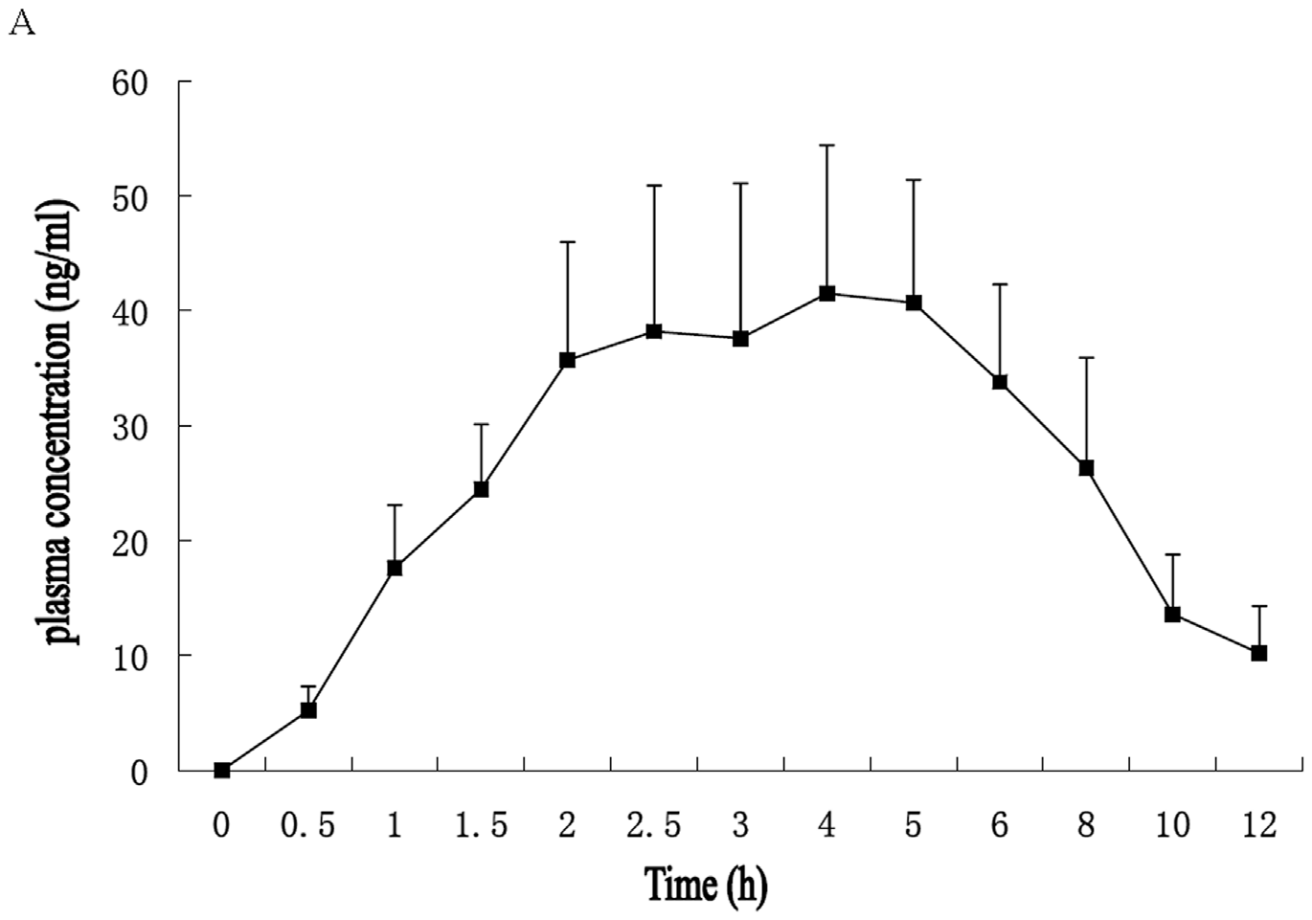
Plasma Concentration of DHC after Oral Gavage in C57BL/6 Mice. (A) Mean plasma concentration–time profiles of DHC after oral gavage (3.0 mg/kg body weight) in 8-week-old C57BL/6 mice. Each point represents mean±S.D. (n = 5).

We also investigated the plasma concentrations of DHC *in vivo* by HPLC as described previously [19]. Mean plasma concentrations in time profiles of DHC after oral gavage (3.0 mg/kg body weight) in 8-week-old C57BL/6 mice were explored. As shown in Fig. 2A, the maximum plasma concentration (C_max_) was 41.5 ng/mL for DHC. The time of maximum plasma concentration (T_max_) was 4 h for DHC (Fig. 2A).

### Effect of DHC on Hepatic Lipid Deposition

**Figure 3:**
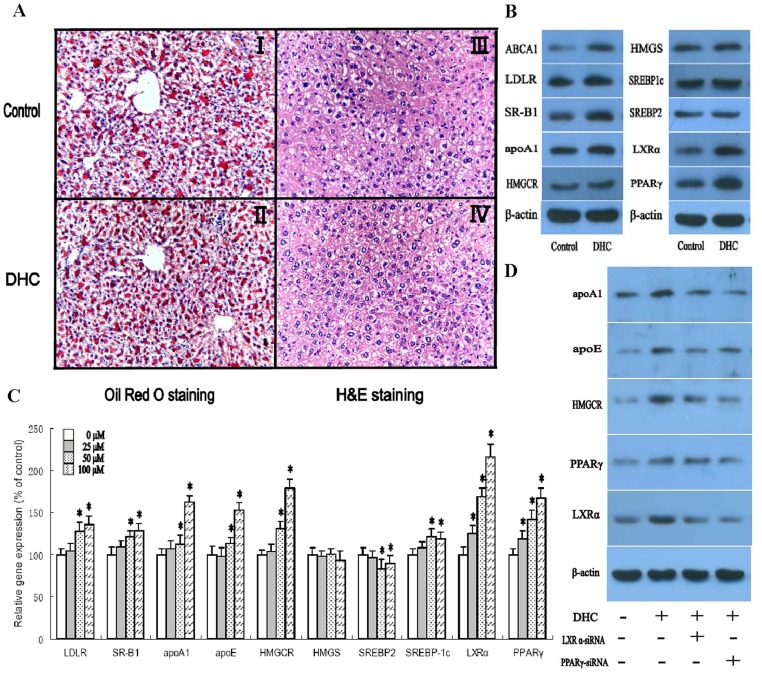
Effect of DHC on hepatic lipid deposition. (A and B) apoE^−/−^ mice were randomized into the control group or the DHC group, and were treated with either vehicle (cholesterol-free vegetable oil) or DHC (3.0 mg/kg body weight) daily by oral gavage for 12 weeks. (I and II), Liver cryo-sections were stained with Oil Red O and hemotoxylin. Original magnification: 100X. (III and IV), Liver paraffin sections were stained with hematoxylin and eosin (H&E). Original magnification: 100X. (B) The protein expression was measured by western blot. (c) HepG2 cells were treated with 0 µM, 25 µM, 50 µM and 100 µM DHC for 24 h. Gene expression was measured by real-time quantitative PCR. (D) The cells were transfected with control or LXRα siRNA or PPARγ siRNA, and then incubated with 100 µM DHC for 24 h. The protein expression was measured by western blot. The results are expressed as the mean ± S.D. of three independent experiments, each performed in triplicate. A and B, the data were compared by unpaired Student's *t* test. C and D, the data were compared by one way ANOVA followed by SNK test. *p<0.05 vs. control group.

The liver is the major organ responsible for the production and degradation of apoB-100-containing lipoproteins [20]. Based on the central role of the liver in determining plasma lipoprotein levels, several therapeutic strategies that act on hepatic lipid metabolism have been developed to reduce the susceptibility to atherosclerosis [21]. Therefore, we next analyzed the effect of DHC on morphology and lipid content in the liver of apoE^−/−^ mice by hematoxylin and eosin (H&E) staining and Oil Red O staining, respectively. Representative images of randomly selected sections of the liver stained for H&E in the control group and the DHC-treated group are shown in Fig. 3A. Based on the number of vacuoles and nuclear size, no notable histological changes in the DHC animals were observed compared with control animals (*p*>0.05). In addition, we found no significant difference in lipid content in the liver between DHC-treated mice and control mice (*p*>0.05).

The protein expression of a series of genes involved in lipid metabolism of mouse liver was investigated by western blot analyses. As shown (Fig. 3B), the DHC group had significantly higher expression of ABCA1, LDLR, SR-B1, apoA1, HMGCR, LXRα and PPARγ than the control group while the DHC group had no expression change of HMGS, SREBP2 and SREBP1c as compared to the control group. Subsequently, expression levels of genes involved in hepatic lipid metabolism in HepG2 cells were analyzed. HepG2 cells were treated with various concentrations of DHC and then analyzed by real-time PCR. As shown in Fig. 3C, DHC treatment slightly reduced gene levels of SREBP2 compared to controls. Moreover, treatment with DHC could up-regulate gene expression of LDLR, SR-B1, apoA1, apoE, HMGCR, SREBP1c, LXRα and PPARγ. In addition, there was no change in gene expression of HMGCS between DHC-treated cells and controls. We then examined the effect of LXRα siRNA and PPARγ siRNA on the regulation of apoA1, apoE, HMGCR, LXRα and PPARγ which was induced by DHC (Fig. 3D). Treatment with both LXRα siRNA and PPARγ siRNA made the up-regulation of DHC on apoA1, apoE and HMGCR expression notably abolished. In addition, treatment with PPARγ siRNA made the DHC-induced up-regulation of LXRα expression notably abolished while treatment with LXRα siRNA had no effect on DHC-induced PPARγ expression in HepG2 cells.

### Effect of DHC on Expression of Genes Involved in Intestinal Lipid Absorption

**Figure 4:**
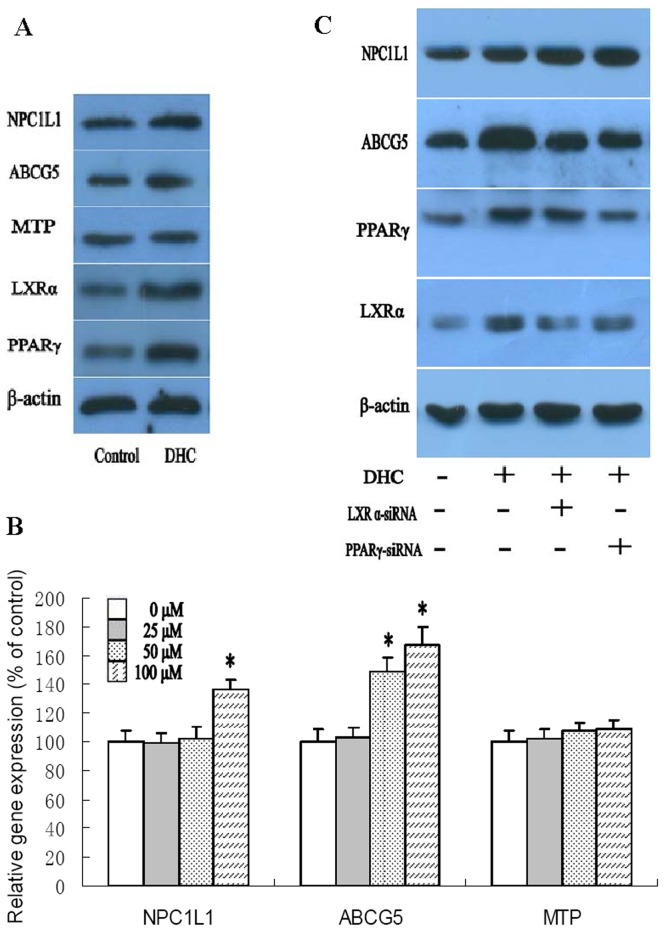
Effects of DHC on expression of genes involved in intestinal lipid absorption. (A) apoE^−/−^ mice were randomized into the control group or the DHC group, and were treated with either vehicle (cholesterol-free vegetable oil) or DHC (3.0 mg/kg body weight) daily by oral gavage for 12 weeks. The protein expression was measured by western blot. (B) Caco-2 cells were treated with 0 µM, 25 µM, 50 µM and 100 µM DHC for 24 h. Gene expression was measured by real-time quantitative PCR. (C) The cells were transfected with control or LXRα siRNA or PPARγ siRNA, and then incubated with 100 µM DHC for 24 h. The protein expression was measured by western blot. The results are expressed as the mean ± S.D. of three independent experiments, each performed in triplicate. A, the data were compared by unpaired Student's *t* test. B and C, the data were compared by one way ANOVA followed by SNK test. *p<0.05 vs. control group.

The definitive identification of intestinal cholesterol transporters and an understanding of their roles in the cholesterol absorption process will be fruitful in improving treatment strategies to suppress cholesterol absorption, thereby reducing hypercholesterolemia and lowering the risk of cardiovascular disease [22]. To investigate the effects of DHC on intestinal cholesterol absorption in apoE^−/−^ mice, protein expression in intestinal tissue of apoE^−/−^ mice were analyzed by western blot (Fig. 4A). We found that DHC could enhance expression of NPC1L1, ABCG5, LXRα and PPARγ while has no effect on MTP expression. In addition, we incubated Caco-2 cells with DHC at various concentrations (Fig. 4B). As shown, treatment with DHC progressively enhanced transcript levels of NPC1L1 and ABCG5. However, we found that there was no significant alteration in MTP gene expression between DHC-treated cells and controls. We then examined the effect of LXRα siRNA and PPARγ siRNA on the regulation of NPC1L1 and ABCG5 which was induced by DHC (Fig. 4C). Treatment with both LXRα siRNA and PPARγ siRNA made the up-regulation of DHC on ABCG5 expression markedly abolished. However, the expression of NPC1L1 significantly increased by treatment with both LXRα siRNA and PPARγ siRNA. Moreover, treatment with PPARγ siRNA made the DHC-induced up-regulation of LXRα expression significantly abolished while treatment with LXRα siRNA had no effect on DHC-induced PPARγ expression in Caco-2 cells.

### Effect of DHC on Plaque Formation

**Figure 5:**
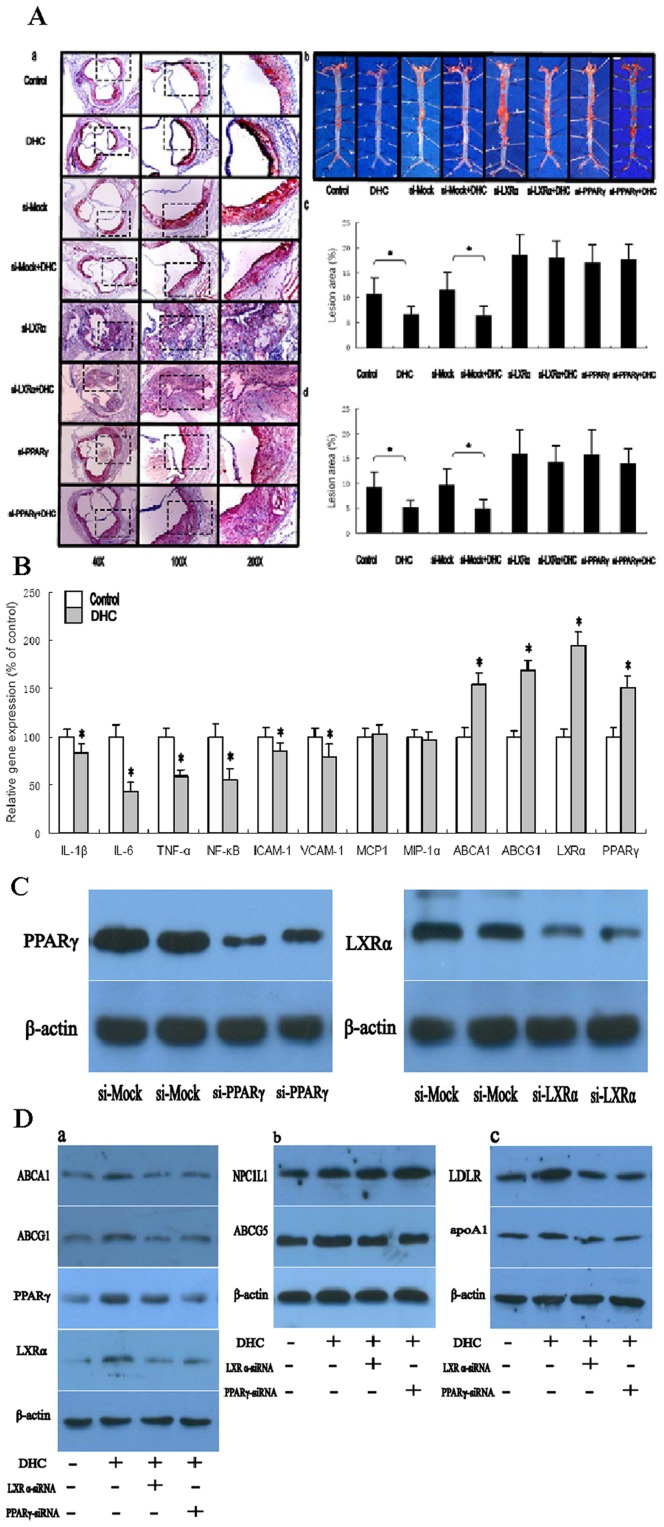
Effect of DHC on atherosclerosis initiation and development in apoE^−/−^ mice. (A and B) apoE^−/−^ mice were randomized into the control group or the DHC group, and were treated with either vehicle (cholesterol-free vegetable oil) or DHC (3.0 mg/kg body weight) daily by oral gavage (0.2 mL per mouse) for 12 weeks. (A) a, Representative staining of aortic valves with Oil Red O. b, Representative staining of aorta with Oil Red O. c, Lesions in aortic valves were analyzed in apoE^−/−^ mice (n = 10 per group, *p*<0.05). d, Lesions of en face preparations were analyzed in apoE^−/−^ mice (n = 10 per group, *p*<0.01). (B) Gene expression was measured by real-time quantitative PCR. Data are expressed as mean values ± S.D. n = 5, performed in triplicate. (C) Western blot analysis of protein levels of LXRα and PPARγ in the aorta in apoE^−/−^ mice treated with control lentvirus (si-Mock) and in apoE^−/−^ mice treated with LXRα siRNA or PPARγ siRNA. Each lane represents an individual animal. Values are mean ± S.D. of three independent experiments. (D) Western blot analysis of protein levels of LDLR, apoA1, LXRα and PPARγ in the liver, ABCG5 and NPC1L1 in the in intestinal tissue, ABCA1 and ABCG1 in the aorta in C57BL/6 mice (WT), and WT mice treated with LXRα siRNA or PPARγ siRNA. Each lane represents an individual animal. Values are mean ± S.D. of three independent experiments. A and D, the data were compared by one way ANOVA followed by SNK test. B and C, the data were compared by unpaired Student's *t* test. *p<0.05 vs. control group.

To investigate the impact of DHC on atherogenesis in apoE^−/−^ mice, atherosclerotic lesions were evaluated by aortic valve section and en face analyses (Fig. 5A). Mice receiving DHC showed a decrease in average lesion area compared with controls by both en face and aortic valve section analyses. Quantification of Oil Red O-stained aortic valve sections revealed that treatment with DHC resulted in a significant 39.3% decrease in lesion area in apoE-deficient mice when compared with controls. To further document the positive effects of DHC on atherosclerosis, Oil Red O-stained lesions in en face preparations of aortas were quantified. Treatment of apoE^−/−^ mice with DHC led to a significant reduction by 44.5% in lesion area compared with controls. In addition, we also investigate the effect of PPARγ and LXRα on DHC-mediated suppression of atherosclerotic lesion formation in vivo by using a lentivirus expressing an siRNA for PPARγ and by an siRNA for LXRα in apoE^−/−^ mice. The protein expression of PPARγ and LXRα in mouse aorta was investigated by western blot analyses. As shown (Fig. 5C), in comparison to the control siRNA, the siRNA of PPARγ suppressed the expression of PPARγ proteins by 75% and the siRNA for LXRα suppressed the expression of LXRα proteins by 81%. Treatment with both LXRα siRNA and PPARγ siRNA made the decrease of atherosclerotic lesion area by DHC markedly abolished.

To explore the mechanisms whereby DHC treatment inhibits plaque progression and stabilization, gene expression changes of the inflammatory factors, adhesion molecules, chemotatic factors, and molecules involved in cellular cholesterol efflux were investigated in the aorta (Fig. 5B). In DHC-treated apoE^−/−^ mice, gene expression of IL-1β, IL-6, TNF-α, ICAM-1, VCAM-1 and NF-κB was markedly repressed but gene expression of ABCA1, ABCG1, LXRα and PPARγ was up-regulated at 12 weeks. In addition, apoE^−/−^ mice, treatment with DHC did not alter gene expression of MCP-1 or MIP-1α.

We subsequently investigated the mechanism of the anti-atherogenic effects of DHC through a PPARγ/LXRα pathway in vivo by using a lentivirus expressing an siRNA for PPARγ and by an siRNA for LXRα in 8-week-old C57BL/6 mice. As shown (Fig. 5D), Treatment with both LXRα siRNA and PPARγ siRNA made the up-regulation by DHC on protein expression of LDLR and apoA1 in the liver, ABCG5 in the in intestinal tissue, ABCA1 and ABCG1 in the aorta markedly abolished. However, the protein expression of NPC1L1 significantly increased by treatment with both LXRα siRNA and PPARγ siRNA. In addition, treatment with PPARγ siRNA made the DHC-induced up-regulation of LXRα expression significantly abolished while treatment with LXRα siRNA have no effect on DHC-induced PPARγ expression *in vivo*.

## Discussion

Coronary atherosclerosis represents the leading cause of morbidity and mortality in men and women throughout the western world [23]. Hypercholesterolemia is a well-established risk factor for the development of atherosclerosis and its pathologic complications. Evidence from clinical trials indicates that reducing plasma cholesterol by dietary and/or pharmacological means leads to a reduction in the incidence of death from cardiovascular disease [24], [25]. In the present study, we showed for the first time that DHC, a major pungent constituent of ‘hot chili peppers’, could decrease lipid content and enhance cholesterol efflux in THP-1 macrophage-derived foam cells. Moreover, DHC markedly decreased plasma levels of LDL-C, VLDL-C, TG and inflammatory cytokines, including IL-1β, IL-6, TNF-α and CRP, and increased plasma levels of HDL-C and apoA1, and thus significantly suppressed atherosclerotic plaque formation in apoE^−/−^ mice fed a high-fat/high-cholesterol diet.

Reverse cholesterol transport (RCT) is a pathway by which accumulated cholesterol is transported from the vessel wall to the liver for excretion, thus preventing atherosclerosis. In atherosclerosis, cellular cholesterol accumulates in lipid-engorged macrophage foam cells and this drives lipid deposition in the atherosclerotic plaque. The control of macrophage cholesterol homeostasis is of critical importance in the pathogenesis of atherosclerosis, as dysregulation of the balance of cholesterol influx, intracellular transport and efflux will lead to excessive accumulation of cholesterol in macrophages and their transformation into foam cells [26]. We observed that DHC exerted an anti-atherogenic effect by decreasing cellular cholesterol content and greatly enhancing expression of NPC1, which is a crucial gene involved in mobilizing cholesterol from intracellular pools to the plasma membrane, and by stimulating expression of ABCA1, ABCG1 and SR-B1, which are key genes involved in mediating the transport of cholesterol across cellular membranes [27]–[29]. We also found that DHC enhanced the overall rate of RCT through the pathways by which HDL-C is delivered to the liver. Mature HDL can transfer its cholesterol to the liver directly via SR-B1 or indirectly via CETP-mediated transfer to apoB-containing lipoproteins, with subsequent uptake by the liver via the LDLR [30]. Here we found that treatment with DHC could up-regulate expression of SR-B1 and LDLR. In addition, the apolipoproteins are important constituents of the plasma lipoproteins and are essential for RCT. ApoA1 is secreted predominantly by the liver and is present on the majority of HDL particles, and its concentrations are closely correlated with plasma HDL [31]. ApoE is a multifunctional protein that plays a key role in the metabolism of cholesterol and triglycerides by binding to receptors on the liver to help mediate clearance of chylomicrons and VLDL from the bloodstream [32]. In the present study, we demonstrated that expression of both apoA1 and apoE were significantly up-regulated by DHC treatment. Consistent with these observations, the plasma levels of TG, LDL-C and VLDL-C were decreased while plasma levels of HDL-C and apoA1 were increased in DHC-treated apoE^−/−^ mice fed a high-fat/high-cholesterol diet. These results provide strong evidence to support the notion that DHC exerts its anti-atherogenic effects by promoting the rate of RCT.

It is known that atherosclerosis is not only a lipid disorder, but also a chronic inflammatory disease. Inflammatory processes take part in all stages of the atherosclerotic process, from lesion initiation to plaque rupture [33], [34]. Macrophages, whether engorged with lipids or not, play a key role in the mediation and modulation of inflammation, and much atherosclerosis research has targeted the role of macrophages in the inflammatory pathways that underlie atherogenesis [35], [36]. In the present study, we showed that DHC significantly down-regulated gene expression of IL-1β, IL-6, TNF-α, CRP and NF-κB in THP-1 macrophage-derived foam cells. To further investigate the mechanisms whereby DHC treatment inhibited plaque progression and stabilization, changes in gene expression of inflammatory molecules were explored in the aorta in apoE^−/−^ mice fed a high-fat/high-cholesterol diet. We found that gene expression of IL-1β, IL-6, TNF-α, and NF-κB were markedly repressed. Consistent with this, the plasma levels of IL-1β, IL-6, TNF-α, and CRP were decreased in DHC-treated apoE^−/−^ mice fed a high-fat/high-cholesterol diet. Our results suggest that DHC-induced suppression effects of inflammatory molecule expression could block or retard the development of atherosclerotic lesions and thus have a positive influence on disease outcomes.

To examine the effects of DHC administration on intestinal lipid absorption, we evaluated expression of NPC1L1, ABCG5 and MTP *in vivo* and *in vitro*. We found that DHC markedly up-regulated the expression of NPC1L1 and ABCG5, while there was no significant alternation in MTP expression. NPC1L1 is present in the brush border membrane of enterocytes in the small intestine and has been identified as an enterotransporter of dietary cholesterol [37]. ABCG5 is an ABC transporter that plays a key role in preventing the intestinal absorption of excess dietary cholesterol from the gut and in enhancing cholesterol efflux from hepatocytes into bile [38]. Thus, our results suggest that intestinal lipid absorption could be regulated by treatment with DHC.

Growing evidence demonstrates that orphan and adopted orphan nuclear receptors, such as liver X receptors and peroxisome proliferator-activated receptors, regulate metabolic profiles in a ligand-dependent or -independent manner in human and animal models [29], [39]. In this study, we showed that gene levels of LXRα and PPARγ were up-regulated by treatment with DHC both *in vivo* and *in vitro*. Our group and others have demonstrated that LXRα could inhibit atherosclerosis initiation and development through regulation of genes involved both in cholesterol elimination and inflammation pathways [17], [40]. It directly induces the expression of ABCA1, ABCG1, SR-B1 and apoE, which mediate cellular cholesterol export in the presence of acceptors, such as HDL and apoA1, and increases the expression of NPC1 and NPC2, which enhance cholesterol trafficking to the plasma membrane [29], [41]–[43]. Previous reports suggest that LXRα can decrease cholesterol absorption by inhibiting NPC1L1 gene expression and inducing ABCG5 gene expression [29]. This indicates that DHC possibly up-regulates the expression of ABCG5 through an LXRα pathway. Interestingly, LXRα not only increases the expression of genes for RCT in macrophages but also independently attenuates the inflammatory response [44]. Activated LXRα could inhibit the activity of NF-κB and its target genes, such as IL-1β and IL-6 in macrophages and in the liver. In addition, the PPARγ has been shown to positively influence several steps of atherogenesis, including inhibiting cell recruitment and activation, decreasing lipid accumulation within the plaque and inhibiting the local inflammatory response through the pathways involved in up-regulating expression of ABCA1, SR-B1, apoA1 and down-regulating expression of VCAM-1, ICAM-1, CRP, IL-1β, IL-6 and TNF-α [45], [46]. In the present study, we showed that treatment with both LXRα siRNA and PPARγ siRNA made the up-regulation of DHC on ABCA1, ABCG1, ABCG5, SR-B1, NPC1, CD36, LDLR, HMGCR, apoA1 and apoE expression notably abolished while making the down-regulation of DHC on SRA1 expression markedly compensated. And treatment with PPARγ siRNA made the DHC-induced up-regulation of LXRα expression and activation notably abolished while treatment with LXRα siRNA had no effect on DHC-induced PPARγ expression and activation. Consequently, these results indicate that DHC may first induce PPARγ expression and activation and then enhance LXRα expression and activation, and the PPARγ/LXRα pathway is involved in DHC-induced enhancement of the rate of RCT and its inhibitory action on proinflammatory genes, and thus are critical for DHC exerting its anti-atherogenic effects. However, whether PPARγ could be directly activated by DHC as a ligand or be indirectly induced by DHC via other signaling pathways need to be further explored.

According to other reports, mice were treated with DHC by the dosage of 3.0 mg/kg body weight daily by oral gavage [8], [47]–[50]. Mean plasma concentrations in time profiles of DHC after oral gavage in mice were explored by HPLC. We found that the maximum plasma concentration (C_max_) was 41.5 ng/mL for DHC. In addition, we found that 100 µM DHC had reasonable effects on cultured cells and performed several in vitro experiments using maximally 100 µM DHC. The plasma concentration of DHC in mice is markedly lower than that in vitro experiment (100 µM DHC). However, a reasonable effect by treatment with DHC was obtained both *in vivo* and *in vitro* experiments according to our results. As we know, the cell transitivity of compounds is often depended on its lipophilicity and the pure DHC is a lipophilic colorless odorless compound. Thus, the cell transitivity may be high *in vivo* than *in vitro*, and that may be one reason to explain the discrepancy of DHC concentration between *in vivo* and i*n vitro*. Further experiments should be carried out to test this presumption.

In conclusion, we have demonstrated that the PPARγ/LXRα pathway is involved in DHC induced decrease of atherosclerotic plaque burden in apoE^−/−^ mice. This beneficial effect was accompanied by a reduction in macrophage-derived foam cell formation, an inhibition of inflammatory gene expression and adhesion molecule expression in the aorta, and the promotion of cholesterol efflux from peripheral tissues. This study provides a new insight into the biological effects and underlying mechanisms for the anti-atherogenic effects of DHC administration.
